# Deep Convolutional Neural Network with Symbiotic Organism Search-Based Human Activity Recognition for Cognitive Health Assessment

**DOI:** 10.3390/biomimetics8070554

**Published:** 2023-11-19

**Authors:** Mohammed Alonazi, Haya Mesfer Alshahrani, Fadoua Kouki, Nabil Sharaf Almalki, Ahmed Mahmud, Jihen Majdoubi

**Affiliations:** 1 Department of Information Systems, College of Computer Engineering and Sciences, Prince Sattam bin Abdulaziz University, Al-Kharj 11942, Saudi Arabia; 2 Department of Information Systems, College of Computer and Information Sciences, Princess Nourah bint Abdulrahman University, Riyadh 11671, Saudi Arabia; 3 Department of Financial and Banking Sciences, Applied College at Muhail Aseer, King Khalid University, Abha 62529, Saudi Arabia; falkoki@kku.edu.sa; 4 Department of Special Education, College of Education, King Saud University, Riyadh 12372, Saudi Arabia; 5 Research Center, Future University in Egypt, New Cairo 11835, Egypt; 6 Department of Computer Science, College of Science and Humanities at Alghat, Majmaah University, Al-Majmaah 11952, Saudi Arabia

**Keywords:** human activity recognition, cognitive health assessment, deep neural networks, hyperparameter tuning, deep convolutional neural network, metaheuristics

## Abstract

Cognitive assessment plays a vital role in clinical care and research fields related to cognitive aging and cognitive health. Lately, researchers have worked towards providing resolutions to measure individual cognitive health; however, it is still difficult to use those resolutions from the real world, and therefore using deep neural networks to evaluate cognitive health is becoming a hot research topic. Deep learning and human activity recognition are two domains that have received attention for the past few years. The former is for its relevance in application fields like health monitoring or ambient assisted living, and the latter is due to their excellent performance and recent achievements in various fields of application, namely, speech and image recognition. This research develops a novel Symbiotic Organism Search with a Deep Convolutional Neural Network-based Human Activity Recognition (SOSDCNN-HAR) model for Cognitive Health Assessment. The goal of the SOSDCNN-HAR model is to recognize human activities in an end-to-end way. For the noise elimination process, the presented SOSDCNN-HAR model involves the Wiener filtering (WF) technique. In addition, the presented SOSDCNN-HAR model follows a RetinaNet-based feature extractor for automated extraction of features. Moreover, the SOS procedure is exploited as a hyperparameter optimizing tool to enhance recognition efficiency. Furthermore, a gated recurrent unit (GRU) prototype can be employed as a categorizer to allot proper class labels. The performance validation of the SOSDCNN-HAR prototype is examined using a set of benchmark datasets. A far-reaching experimental examination reported the betterment of the SOSDCNN-HAR prototype over current approaches with enhanced precision of 86.51% and 89.50% on Penn Action and NW-UCLA datasets, respectively.

## 1. Introduction

Cognitive impairment is a brain condition arising from trauma like road accidents or sports injuries, old age, or other reasons, which include inflammatory or vascular insults. Certain signs of cognitive impairment are memory concerns or other cognitive complaints [1]. Non-memory triggers have changes in personality, depression, and worsening of chronic disease and balance or fall problems. Elderly persons, individuals with dementia, and people with mild cognitive impairment require permanent observation by a nurse or doctor every time [2]. Thus, there is a necessity for automatic and sustainable resolutions for assessing the health condition of a person efficiently and rapidly [3]. Human action recognition (HAR) is a crucial perspective of people-to-people interaction since this offers information regarding the nature of humans. For instance, a person’s identity, personality, and mental condition become tough to derive. In recent times, there has been a rise in deaf–mute people [4]. It is understood that deaf–mute people are not able to communicate with non-deaf–mute people, whereas non-deaf–mute people do not understand the meanings of gestures.

Human action popularity makes for a pleasant verbal interchange platform for such humans to interact with non-deaf–mute persons [5]. In this study, cognizance in vision was primarily related to a hand reputation technique that was larger natural and convenient for informative hand gestures. Many feature extraction policies exist as well as categorization policies. Among them, selecting appropriate strategies for usage becomes a complicated problem [6]. The most significant methodology was segmentation; in this method, the foreground was detached from the background [7]. This separation demands feature extraction approaches for computation, like angle computation, accuracy calculation, and outcome estimation. In this video, in action detection, each hand movement has a distinct purposeful text format [8]. It can be slightly different from still images, due to human activities that have a collection of moving components or ever-changing motions [9]. Therefore, finding meaningful text for a suitable gesture becomes significant. So, it is necessary to scrutinize changing spatial–temporal highlights for action acknowledgement [10].

HAR serves as a core technology to improve various aspects of day-to-day life, including healthcare monitoring and assisting individuals with physical disabilities. By automating the identification and classification of human behaviors and actions, it allows for the development of ground-breaking applications that improve health, safety, and overall quality of life. Deep learning (DL) techniques are instrumental in human activity recognition due to their ability to efficiently analyze and process complex sequences and patterns in sensor data [11]. With the abundance of sensor-generated data and the proliferation of wearable devices, DL techniques can discern intricate features in motion and physiological signals, enabling accurate detection and classification of human activities [12,13]. These models have the potential to improve applications in sports analysis, healthcare, security, and many other fields, offering a more versatile and sophisticated approach to understanding human behavior and movement patterns.

This research develops a novel Symbiotic Organism Search with a Deep Convolutional Neural Network-based Human Activity Recognition (SOSDCNN-HAR) model for Cognitive Health Assessment. The goal of the SOSDCNN-HAR model is to recognize human activities in an end-to-end way. For the noise elimination process, the presented SOSDCNN-HAR model involves the Wiener filtering (WF) technique. In addition, the presented SOSDCNN-HAR model follows a RetinaNet-based feature extractor for automated extraction of features. Moreover, the SOS procedure is exploited as a hyperparameter optimizing tool to enhance recognition efficiency. Furthermore, the Gated Recurrent Unit (GRU) prototype can be employed as a categorizer to allot proper class labels. The performance authentication of the SOSDCNN-HAR prototype is examined by implementing a set of benchmark databases.

In short, the influence of the research is detailed in the following.

- Develop a new SOSDCNN-HAR prototype for activity recognition and categorization.
- Implement WF-based preprocessing and RetinaNet-based feature-extracting process to produce feature vectors.
- Present the SOS algorithm as a hyperparameter-optimizing tool to enhance the recognition efficiency of the RetinaNet prototype.
- Employ the GRU classification model for accurate and proficient classification of human activities.

The remaining sections of the study are provided in the subsequent sections. The existing HAR prototypes are provided in Section 2, and the suggested prototype is elaborated on in Section 3. Then, Section 4 bestows performance authentication, and Section 5 concludes the study.

## 2. Related Works

The authors in [14] concentrate on the DL-precipitated HAR in an IoHT atmosphere. A semi-supervised DL construction was developed and constructed to further accurate HAR that efficaciously investigated and implemented the feebly tagged sensor dataset in training the categorization learning models. To resolve the challenge of the insufficiently tagged trials, a smart automatic labelling structure relying upon a Deep Q-Network (DQN) was constructed by a recently developed distance-based rewarding rule that could precipitate learning effectiveness in the IoT atmosphere. Hassan et al. [15] presented a smartphone inertial node-founded technique for HAR. Firstly, an efficient feature is extracting raw information. The feature includes mean, median, autoregressive coefficients and more. Furthermore, the feature is processed by employing an LDA and kernel PCA (KPCA) to make them very powerful. At last, the feature is trained by a Deep Belief Network (DBN) for effective HAR.

Gumaei et al. [16] introduced robust multiple node-based architectures for HAR with a fusion DL technique that incorporated Simple Recurrent Units (SRUs) with the GRU of a NN. The study utilized a deep SRU for processing the sequence of multiple modal input datasets by utilizing the ability of the memory’s internal state. Moreover, the researcher used a deep GRU to learn and stock the historical data to be sent to the upcoming state for resolving instability or fluctuation in precision and gradient-disappearing issues. Mukherjee et al. [17] developed a group of three classifier techniques, CNN-LSTM, CNN-Net, and Encoded-Net, i.e., called EnsemConvNet. All of this categorization model is founded on simple 1D-CNN; however, it is diverse in terms of other key alterations from the infrastructure, kernel size, and number of dense layers. All the models accept the time sequence information as a 2D matrix by taking a window of information at a time for inferring records that ultimately forecast the kinds of human activities.

Abdel-Basset et al.’s [18] project was a supervised dual-channel prototype that encompassed attention and LSTM models for temporal integration of an inertial sensor dataset coexisting with convolution ResNet for spatial integration of the sensor dataset. Likewise, the scientists presented an adaptive channel-squeezing process for fine-tuning CNN feature extraction capability by using multi-channel dependencies. Zahin et al. [19] developed a new method using CNN by varying kernel dimensional and BiLSTM to take aspects at diverse resolutions. The innovation of this research exists within the effective selection of optimal video depiction and extracts spatial and sequential characteristics from sensory information by making use of BiLSTM and conventional CNN. Though the existing works exploit DL models for HAR, most of the works do not focus on the hyperparameter optimization process. Therefore, in this work, the hyperparameter tuning technique is achieved by the usage of the SOS procedure.

In [20], the authors considered DL-improved HAR in IoHT platforms. A semisupervised DL approach was developed and made for highly accurate HAR that proficiently implemented and examined the weakly labelled sensor data for training the classifier learning method. In [21], an efficient technique was introduced that could recognize human activities in videos utilizing a single decisive pose. For accomplishing the task, a decisive pose was removed, employing optical flow, and then feature extraction was achieved by a two-fold transformation of wavelet. The two-fold transformation could be attained through Ridgelet Transform (RT) and Gabor Wavelet Transform (GWT). Tan et al. [22] developed an ensemble learning algorithm (ELA) to execute activity identification employing the signals recorded via smartphone sensors. This developed ELA incorporated a gated recurrent unit (GRU), a CNN stacked with the deep neural network (DNN), and a GRU.

Most of the existing HAR techniques do not focus on the hyperparameter selection approach, which mostly affect the performance of classification algorithm. Especially, the hyperparameters, including batch size, epoch count, and learning rate selection, are crucial to obtain effectual outcome. As the trial-and-error model for hyperparameter tuning is an erroneous and tedious process, metaheuristic algorithms can be applied. Thus, in this work, we apply the SOS algorithm for the parameter selection of the RetinaNet model.

## 3. The Proposed Model

**Figure 1:**
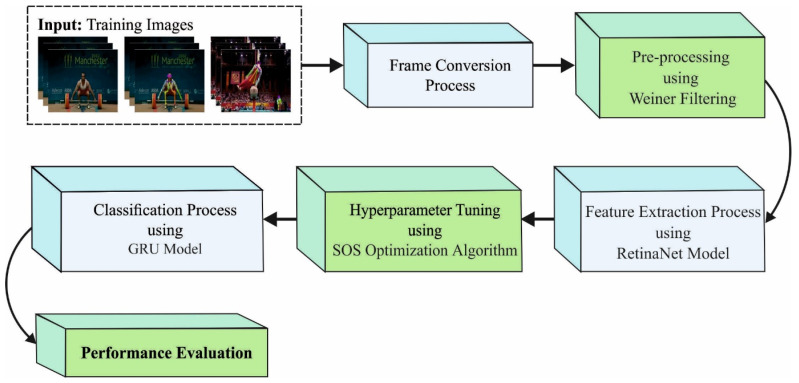
Block diagram of the SOSDCNN-HAR procedure.

In this research, a novel SOSDCNN-HAR approach was presented for the automatic recognition of the actions of humans. Figure 1 shows the block diagram of the SOSDCNN-HAR procedure. It includes a series of processes, namely, frame conversion, WF-based preprocessing, RetinaNet feature extraction, SOS-based hyperparameter tuning, and GRU-based classification. Primarily, the proposed model enables the frame conversion process, where the videos are transformed into a set of frames. The proposed SOSDCNN-HAR model applied the WF-based noise elimination method to eradicate the noise. Additionally, the RetinaNet-based feature extractor and SOS-based hyperparameter optimizer are applied. In addition, the GRU prototype is enforced for the process of categorization.

### 3.1. Image Pre-Processing

Firstly, the suggested SOSDCNN-HAR prototype was initially applied to the WF-founded noise removal approach to eliminate the noise. The Wiener function uses a WF (variety of linear filtering) to an image adaptively, modifying itself to local image discrepancy. When it is small, the Wiener carries out further smoothing. Once the variance is larger, the Wiener carries out a little smoothing. This technique frequently generates good outcomes when compared to linear filtering. The adaptive filter was additionally selective when compared to a comparable linear filter, which preserved edge, and another higher frequency part of the image. Additionally, there were no tasks for this design; the Wiener2 function managed each primary computation and performed the filter for the input images. It necessitated additional computational time when compared to linear filtering. Wiener executes as superior if the noising is constant-power (“white”) additive noising, namely, Gaussian noise.

### 3.2. RetinaNet-Based Feature Extractor

After pre-processing the imagery, the RetinaNet-founded factor extractor and SOS-based hyperparameter optimizer are applied. RetinaNet comprises a Feature Pyramid Network (FPN), ResNet, and two Full Convolution Networks (FCNs) [23]. ResNet employs a dissimilar network layer. A significant part of ResNet is the idea of RL that allows raw input datasets to be transmitted to the following levels. Select 101 layers with optimum training efficacy. Next, extract the features of echocardiography using ResNet and then transfer them to the subsequent subnetwork. FPN is a technique to efficiently extract the features of every dimension in an image using a CNN. Initially, employ single dimensional image as the input to ResNet. Then, starting from other layers of the convolutional networks, the features of every layer are designated using the FPN and then incorporated to generate the concluding outcome. The class subnets in the FCN execute the categorizer technique. The focal loss can be a revised edition of the binary and cross-entropy form given by:

(1) $$CE\left(p,\;y\right)=\left\{\begin{array}{cc} -\;\log\;\left(p\right), & if\;y=1,\\ -\;\log\;\left(1-p\right), & or\;else, \end{array}\right.$$

where $y\in \left[\pm ,\;1\right]$ describes the ground truth, and $p\in \left[0,\;1\right]$ indicates the contemplation possibility of technique for y=1.

(2) $$p_{t}=\left\{\begin{array}{cc} p, & if\;y=1,\\ 1-p, & otherwise, \end{array}\right.$$

The aforementioned formula is expressed by

(3) $$CE\left(p,\;y\right)=CE\left(p_{t}\right)=-\;\log\;\left(p_{t}\right)\;.$$

To overcome the challenge of the dataset imbalance amongst the positive and negative examples, the novel version is transformed into a succeeding form:

(4) $$CE\left(p_{t}\right)=-\alpha_{t}\;\log\;\left(p_{t}\right),\;$$

Amongst them,

(5) $$\alpha_{t}=\left\{\begin{array}{ll} \alpha , & ify=1\\ l-\alpha , & otherwise \end{array}\right.\;$$

Whereas $\alpha \in \left[0,1\right]$ describes the weight factor. To overcome the shortcomings, the C variable was projected to attain the concluding procedure of focal loss.

(6) $$FL\;\left(p_{t}\right)=-\alpha_{t}{\left(1-p_{t}\right)}^{\gamma }\;\log\;\left(p_{t}\right).\;$$

### 3.3. Hyperparameter Optimization

At this stage, the SOS algorithm is applied to regulate the hyperparameters associated with the RetinaNet prototype. SOS is nature inspired, population based, and benefits from randomness to some degree [24]. Two kinds of symbiotic connections might exist among any two different organisms: facultative or compulsory. Initially, the existence of two species is based on one another, whereas, in the last case, two species could non-essentially cohabitate in commonly advantageous relationships. In SOS, the searching technique was initialized using an N-random population. Next, the population member is enhanced by utilizing three real-time symbiotic stages: parasitism, mutualism, and commensalism.

#### 3.3.1. Mutualism Phase

This SOS stage reproduces the mutualistic relationships that are beneficial for organisms, i.e., all the organisms are affected positively by other activities. Assume $X_{i}$ as $i^{th}$ organisms and $X_{j}$ as an arbitrarily chosen organism where j≠i, and use mutualistic sense to improve the existence probability in $an_{}$ ecosystem. Consequently, novel trial solutions $X_{inew}$ and $X_{jnew}$ are evaluated by the following expression, and it is replaced by $X_{j}$ and $X_{j}$ if the fitness value is more efficient.

(7) $$X_{inew}=X_{j}+rand\left(0,\;1\right)\times \left(X_{best}-Mutual_{-}Vector\times BF1\right)$$

(8) $$X_{jnew}=X_{j}+rand\left(0,\;1\right)\times \left(X_{best}-Mutual_{-}Vector\times BF2\right)$$

(9) $$M\iota ltllal\_-Vector=\frac{X_{i}+X_{\dot{j}}}{2}$$

In Equations (7) and (8), rand $\left(0,\;1\right)$ returns an arbitrary number in the uniform distribution within $\left[0,\;1\right],$ $X_{best}$ denotes the ecosystem optimal organism, and *BF*1 and *BF*2 benefit factors arbitrarily allocated to 1 (partially beneficial) or 2 (fully beneficial), which determine the degree of advantage to all the organisms. The balance between exploitation and exploration largely depends on an arbitrary value of the benefit factor.

#### 3.3.2. Commensalism Phase

In the commensalism stage, a single organism attains benefits, whereas the rest is not impacted by the engagement either positively or negatively. Similar to the mutualism stage, $X_{i}$ is arbitrarily chosen. Now, $X_{i}$ indicates the organism whose aim is to benefit from the interaction, whereas $X_{j}$ denotes the neutral one, insensitive to relationship types. The novel experimental outcome $X_{inew}$ is computed, and the process is forwarded using $X_{inew}$ when it is superior to $X_{i}.$

(10) $$X_{inew}=X_{i}+rand\left(-1,\;1\right)\times \left(X_{best}-X_{j}\right)\;$$

It can be noted from Equation (10) that the novel trial organism is attained according to the difference $X_{best}-X_{j}$ multiplied using a random value, compared to −1 and 1, to extend the searching space compared to the rand (0, 1).

#### 3.3.3. Parasitism Phase

It is a type of symbiotic association in which a single organism, such as a parasite, adapts for sustenance by benefiting from other organisms, such as a host, which causes minor damage. Here, the artificial parasite organism named $Parasite_{-}$*Vector* was generated by altering and duplicating arbitrary elements of $X_{i}$ with arbitrary values from the lower LB and upper UB search boundary. Next, an organism $X_{j}$ is allocated as a host organism to parasites. The organism tries to eliminate one another, and the one with the best fitness value will destroy the other one and defeat their location in the ecosystem.

(11) $$Parasite_{-}Vector=\left\{\begin{array}{ll} X_{i}^{d} & if\;rand\;\left(0,\;1\right)<rand\left(0,\;1\right)\\ LB+rand\left(0,\;1\right)\times \left(UB-LB\right) & else \end{array}\right.\;$$

where $X=\left[X^{1},X^{2},\ldots ,X^{D}\right]$, and D implies the design variable count.

The parasitism stage presents random variations in the ecosystem by protecting organisms in local minimal stagnation, and therefore it acts as a major component in fulfilling the exploration ability of the technique or the global search performance.

Fitness selection is a considerable factor that influences the performance of the SOS approach. The hyperparameter selection process involves the solution encoding approach to evaluate the efficacy of the candidate solutions. In this work, the SOS algorithm considers accuracy as the major criterion to design the fitness function, which can be formulated as follows.

Fitness = max (*P*)

(12) $$P=\frac{TP}{TP+FP}\;$$

From the expression, *TP* represents the true positive, and *FP* denotes the false positive value.

### 3.4. GRU Based Categorization

In the last stage, the GRU prototype can be implemented as a categorizer when allocating proper class tags. A GRU is an enhanced version of a typical RNN and is a basic version of LSTM. Therefore, a GRU is different from LSTM, and, sometimes, it can generate similarly outstanding outcomes. Related to the LSTM, a GRU was planned to adjustably reset/update its memory. Therefore, the GRU is a reset and update gate that is the same as forgetting and input gates from LSTM. But, the GRU completely depicts its memory contents from the always step and also balances it among the prior and new content of the memory, utilizing leaky combination organized by gate updating. The GRU infrastructure is the same as the LSTM framework, as some parameters allow it to simply capture long-term dependency further. The update gate monitors the count of memory contents, which is essential to be forgotten in the preceding time stages. In addition, it controls the count of memory contents, which is necessary to add in the present time step. Equation (13) calculates this performance.

(13) $$z_{n}=\sigma \left(W_{z}\;\left[h_{n-1},x_{n}\right]\right)$$

The method utilizes the reset gate for determining the count of records for forgets, as provided in Equation (14).

(14) $$r_{n}=\sigma \left(W_{r}\;\left[h_{n-1},x_{n}\right]\right)$$

A novel memory content was established by employing the reset gate computed in Equation (14), and associated past data were saved as illustrated in Equation (15).

(15) $$\hat{h}=tanh\left(W\cdot \left[r_{n}\times h_{n-1},x_{n}\right]\right)$$

Lastly, the network computes the concealed state $h_{n}$, which is a vector that transmits data to the present unit and permits it down to networking. Hence, the update gate was important, as it chose the needful in the present memory content ${\hat{h}}_{n}$ and the preceding step $h_{n-1}$. Equation (16) computes the value of $h_{n}$.

(16) $$h_{n}=\left(1-z_{n}\right)\times h_{n-1}+z_{n}\times \hat{h}\;$$

Therefore, GRUs save and filter the data, employing its upgrade and reset gates, creating them as a chosen special when trained suitably.

## 4. Experimental Validation

The suggested prototype was duplicated by employing the Python 3.6.5 tool. The suggested procedure was evaluated on with 16 GB RAM, a 250 GB SSD, an i5-8600k CPU, a GeForce 1050Ti 4 GB, and a 1 TB HDD. The parameter setups were specified as follows: learning rate: 0.01; dropout: 0.5; batch size: 5; epoch count: 50; and activation: ReLU.

This section validates the achievement of the SOSDCNN-HAR prototype on three distinct datasets: the UCF-Sports Action Dataset [25], the Penn Action dataset [26], and the NW-UCLA dataset [27]. The first dataset, the UCF-Sports Action Dataset, held samples under distinct activities, such as Upper Head (H), Left Shoulder (LS), Left Hand (LD), Right Shoulder (RS), Right Hand (RD), Right Hip (RH), Left Hip (LH), Right Knee (RK), Left Knee (LK), Left foot (LF), Right foot (RF), and Torso (T).

### 4.1. Result Analysis on UCF-Sports Action Dataset

Table 1 offers a detailed accuracy investigation of the SOSDCNN-HAR prototype with recent prototypes on the trial UCF-Sports Action Dataset [28,29,30]. The outcomes indicated that the SVM and DT prototypes resulted in lower precision percentages of 77.81% and 78.18%, respectively. The CNN model reached moderate accuracy of 83.65%. After that, the APAR-MMSHF prototype accomplished a slightly precipitated accuracy of 89.31%. However, the SOSDCNN-HAR model attained a maximum accuracy of 93.52%.

**Figure 2:**
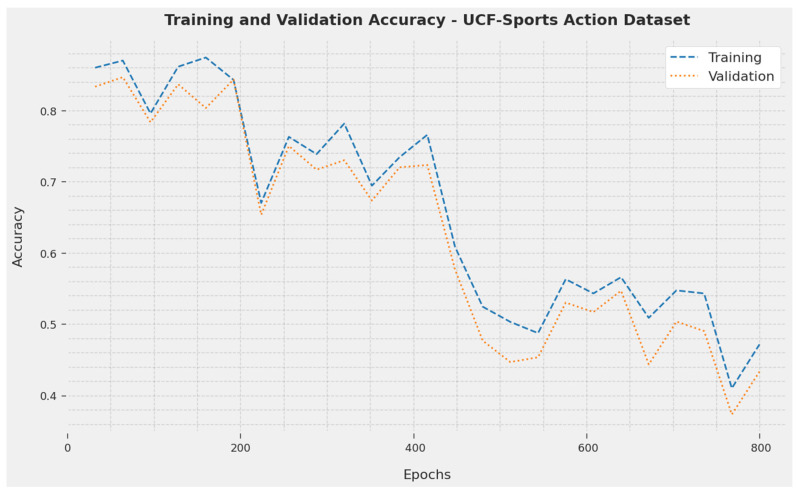
TA and VA evaluations of the SOSDCNN-HAR technique on the UCF-Sports Action Dataset.

The Training Accuracy (TA) and Validation Accuracy (VA) achieved by the SOSDCNN-HAR technique on the UCF-Sports Action Dataset are shown in Figure 2. The investigational result inferred that the SOSDCNN-HAR procedure attained maximal VA and TA values. In particular, the VA appeared to be greater than TA.

**Figure 3:**
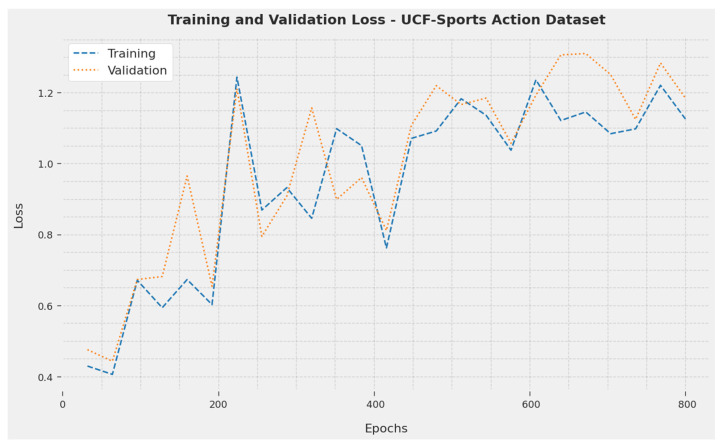
TL and VL examination of SOSDCNN-HAR procedure on UCF-Sports Action Dataset.

The Training Loss (TL) and Validation Loss (VL) accomplished by the SOSDCNN-HAR approach on the UCF-Sports Action Dataset are recognized in Figure 3. The investigational result implied that the SOSDCNN-HAR algorithm achieved minimum values of TL and VL. In particular, the VL was lesser than the TL.

**Figure 4:**
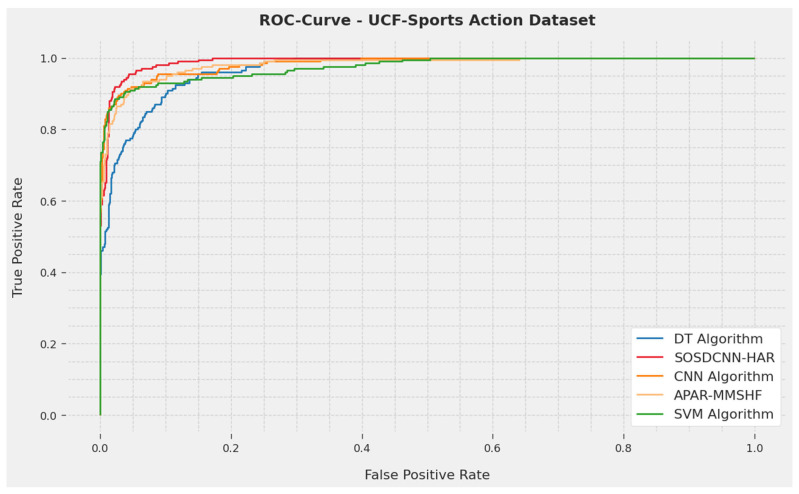
ROC curve evaluation of the SOSDCNN-HAR procedure on the UCF-Sports Action Dataset.

A short-term ROC analysis of the SOSDCNN-HAR technique on the UCF-Sports Action Dataset is depicted in Figure 4. The results show that the SOSDCNN-HAR technique showed its capability in classifying diverse techniques on the UCF-Sports Action dataset.

### 4.2. Result Analysis on Penn Action Dataset

Table 2 provides a detailed precision examination of the SOSDCNN-HAR methodology with the current prototypes on the test Penn Action Dataset. The outcomes depict that the PAAP and JAR-PSV procedures have the capabilities to reduce the accuracies by 78.93% and 86.51% subsequently. Next, the BJG-3D Deep Conv approach acquired a moderate accuracy of 97.83%, followed by the BEP and ARC-VAPS approaches that accomplished slightly enhanced exactness percentages of 98.74% and 98.92% subsequently. However, the SOSDCNN-HAR procedure attained a maximum precision of 99.01%.

**Figure 5:**
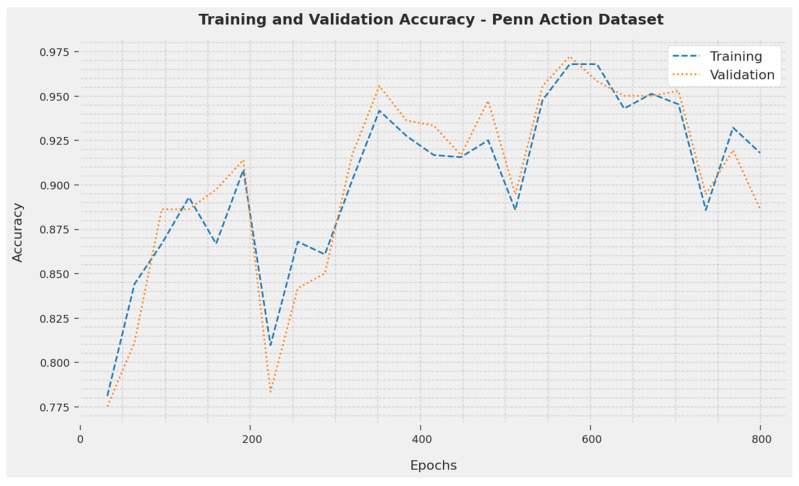
TA and VA examination of the SOSDCNN-HAR approach on the Penn Action Dataset.

The TA and VA acquired by the SOSDCNN-HAR technique on Penn Action Dataset are portrayed in Figure 5. The investigational result denoted that the SOSDCNN-HAR approach attained extreme TA and VA values. Exactly, the VA was larger than the TA.

**Figure 6:**
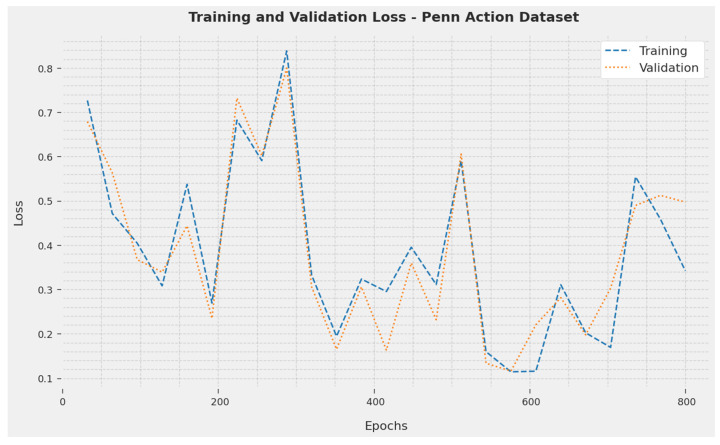
TL and VL examination of SOSDCNN-HAR procedure on Penn Action Dataset.

The TL and VL obtained by the SOSDCNN-HAR algorithm on the Penn Action Dataset are recognized in Figure 6. The investigational result concluded that the SOSDCNN-HAR methodology has achieved minimum values of TL and VL. Especially, the VL appeared to be lesser than the TL.

**Figure 7:**
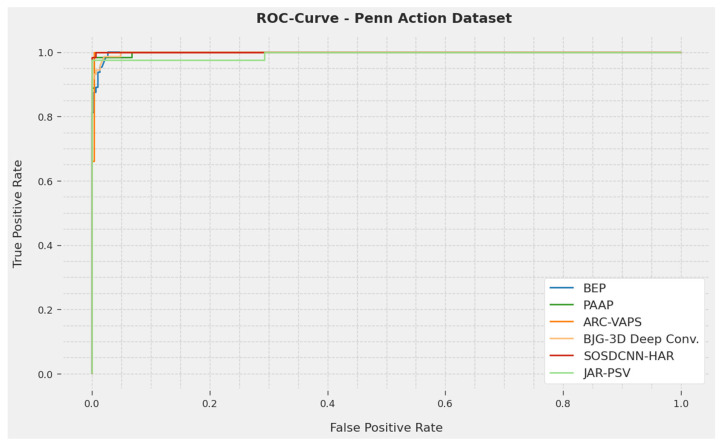
ROC curve examination of the SOSDCNN-HAR approach on the Penn Action Dataset.

An elaborated ROC study of the SOSDCNN-HAR procedure on the Penn Action Dataset is illustrated in Figure 7. The outcomes signify that the SOSDCNN-HAR procedure revealed its competence in categorizing diverse methods in the Penn Action dataset.

### 4.3. Result Analysis on NW-UCLA Dataset

Table 3 presents an elaborated precision examination of the SOSDCNN-HAR approach with recent prototypes on the trial NW-UCLA Dataset. The resulting data showed that the ETESPT-HAR and PCSTA-HAR methods had the capabilities for less precision percentages of 75.76% and 85.01%, respectively. Subsequently, the BVA technique achieved a moderate accuracy of 87.29%. Next, the BEP and ARC-VAPS methods established slightly enhanced accuracies of 87.78% and 88.21%, respectively. However, the SOSDCNN-HAR algorithm gained an extreme accuracy of 89.50%.

**Figure 8:**
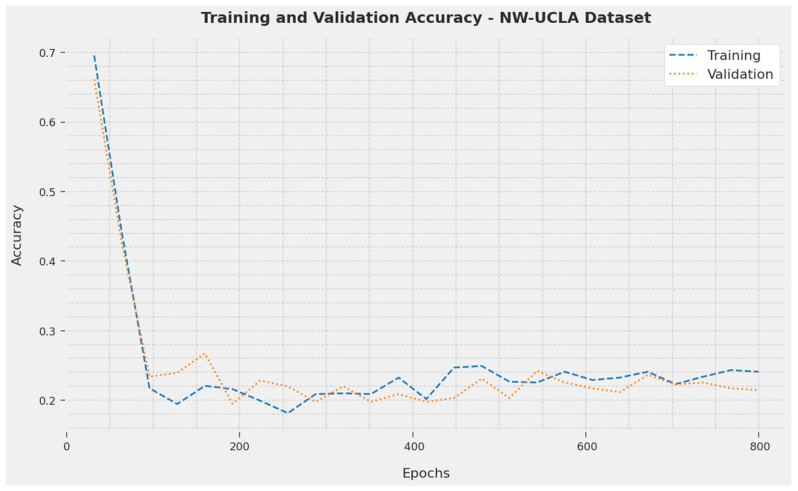
TA and VA examination of the SOSDCNN-HAR technique on the NW-UCLA Dataset.

The TA and VA acquired by the SOSDCNN-HAR method on the NW-UCLA Dataset are depicted in Figure 8. The investigational result implicated that the SOSDCNN-HAR technique achieved the greatest values of TA and VA. In particular, the VA was greater than the TA.

**Figure 9:**
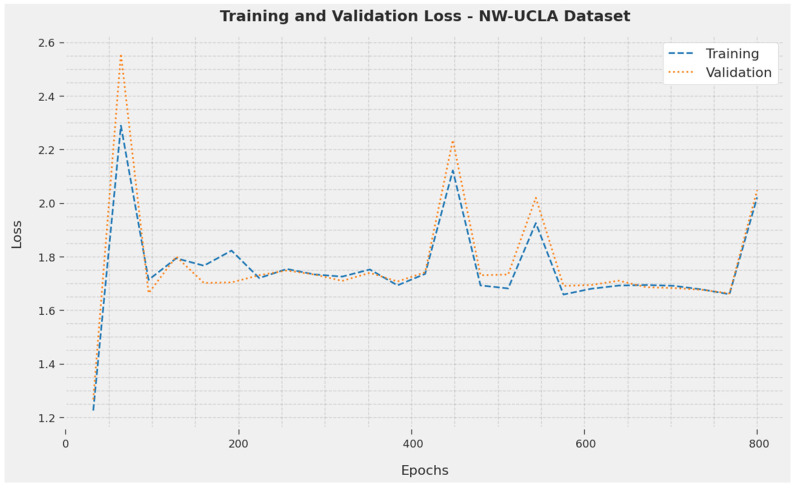
TL and VL examination of the SOSDCNN-HAR approach on the NW-UCLA Dataset.

The TL and VL reached by the SOSDCNN-HAR procedure on the NW-UCLA Dataset are recognized in Figure 9. The investigational outcome concluded that the SOSDCNN-HAR procedure achieved minimum values of TL and VL. To be precise, the VL was lesser than the TL.

**Figure 10:**
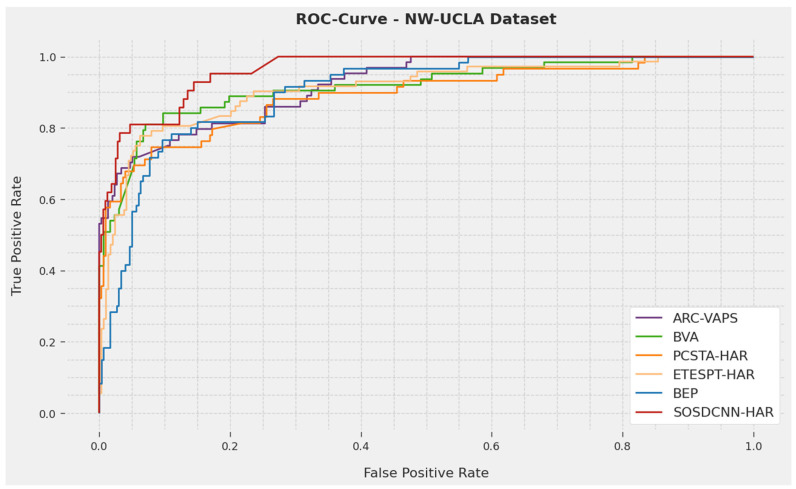
ROC curve analysis of the SOSDCNN-HAR approach on the NW-UCLA Dataset.

A short ROC examination of the SOSDCNN-HAR procedure on the NW-UCLA Dataset is represented in Figure 10. The resulting data depicted that the SOSDCNN-HAR algorithm exhibited its capacity to classify diverse approaches on the NW-UCLA dataset. Therefore, it is apparent that the proposed prototype can recognize diverse human activities.

The higher performance of the SOSDCNN-HAR model over current methods can be attributed to its complete technique that integrates deep convolutional neural networks with human activity detection. This combination permits end-to-end detection of human activities with improved noise removal over Wiener filtering, automated feature extraction employing RetinaNet, and efficient hyperparameter optimization through the SOS process. These united powers result in significantly enhanced precision, showcasing the technique’s capacity to beat the present techniques and provide robust solutions for cognitive health assessments. Hyperparameter optimization via the SOS process fine-tunes the model’s settings, ensuring that it works at peak efficacy. This particular technique of parameter tuning is vital in reaching the excellent precision reported in the experimental outcomes.

## 5. Conclusions

In the presented research, a novel SOSDCNN-HAR procedure was established for the recognition of the actions of an individual automatically. It incorporates several subprocesses, such as WF-based preprocessing, RetinaNet feature extraction, SOS-based hyperparameter tuning, and GRU-based classification. The SOSDCNN-HAR technique is validated on three distinct datasets: the UCF-Sports Action Dataset, the Penn Action dataset, and the NW-UCLA dataset. The SOSDCNN-HAR model developed a promising solution for cognitive health assessment, leveraging deep convolutional neural networks and human activity detection. The model’s robust noise recognition via Wiener filtering, feature extraction utilizing RetinaNet, and hyperparameter optimization via SOS development collectively contribute to its outstanding performance. With exciting exactness levels attained on benchmark datasets, the SOSDCNN-HAR technique displays its possibilities to advance the field of cognitive health assessment and provide valuable insights for medical and study applications. Upcoming work for the SOSDCNN-HAR model concentrates on many key avenues of enhancement and survey. Primarily, a model can be prolonged to provide a wide variety of human activities, confirming its applicability in a broader spectrum of scenarios. In addition to that, the incorporation of multi-modal data sources, namely, sensor data and wearable technology, improves the model’s accuracy and offers a more holistic view of cognitive health. Lastly, real-world deployment and validation of the SOSDCNN-HAR model will be critical to evaluate its performance and usability in experimental and healthcare settings.

## Figures and Tables

**Table 1:** Accuracy analysis of SOSDCNN-HAR approach with recent algorithms on the UCF-Sports Action Dataset.

Methods	Accuracy (%)
SVM Algorithm	77.81
DT Algorithm	78.18
CNN Algorithm	83.65
APAR-MMSHF	89.31
SOSDCNN-HAR	93.52

**Table 2:** Accuracy examination of SOSDCNN-HAR procedure with recent methods on Penn Action Dataset.

Methods	Accuracy (%)
JAR-PSV	86.51
PAAP	78.93
BJG-3D Deep Conv.	97.83
BEP	98.74
ARC-VAPS	98.92
SOSDCNN-HAR	99.01

**Table 3:** Accuracy examination of SOSDCNN-HAR technique with recent procedures on NW-UCLA Dataset.

Methods	Accuracy (%)
ETESPT-HAR	75.76
PCSTA-HAR	85.01
BVA	87.29
BEP	87.78
ARC-VAPS	88.21
SOSDCNN-HAR	89.50

## Data Availability

Data sharing does not apply to this article, as no datasets were generated during the current study.
